# CAG Postdoctoral – A2 CROHN DISEASE EXCLUSION DIET MAINTENANCE THERAPY INDUCES SUSTAINED MICROBIOME CHANGES

**DOI:** 10.1093/jcag/gwaf042.002

**Published:** 2026-02-13

**Authors:** A Armet, R Sigall-Boneh, Y Zhao, L Luo, V Navas-López, S Hussey, G Pujol Muncunill, S Lawrence, H Jonsson Rolandsdotter, A Otley, J Martín-de-Carpi, F Li, J van Limbergen, E Wine

**Affiliations:** Pediatrics, University of Alberta, Edmonton, AB, Canada; Edith Wolfson Medical Center, Holon, Tel Aviv District, Israel; Zhejiang University, Hangzhou, Zhejiang, China; Zhejiang University, Hangzhou, Zhejiang, China; Hospital Regional Universitario de Malaga, Málaga, AL, Spain; Royal College of Surgeons in Ireland, Dublin, Leinster, Ireland; Sant Joan de Déu Barcelona, Barcelona, Spain; BC Children’s Hospital, Vancouver, BC, Canada; Karolinska Institutet, Stockholm, Stockholm County, Sweden; IWK Health Csssentre, Halifax, NS, Canada; Sant Joan de Déu Barcelona, Barcelona, Spain; Zhejiang University, Hangzhou, Zhejiang, China; Amsterdam Universitair Medische Centra, Amsterdam, NH, Netherlands; SickKids Research Institute, Toronto, ON, Canada

## Abstract

**Background:**

Crohn disease (CD) exclusion diet plus partial enteral nutrition (CDED+PEN) and exclusive enteral nutrition (EEN) are both effective at inducing remission in pediatric CD.

**Aims:**

The aims of the current study were to characterize temporal microbiome alterations in children with CD receiving either CDED+PEN or EEN, and determine if microbiome restoration (more closely resembling healthy controls) was associated with sustained remission. We hypothesized that the diet therapies would induce distinct changes to the gut microbiome, and that this would be linked to improved clinical outcomes.

**Methods:**

In a multi-centre randomized controlled trial, children (12.7±2.4 years) with mild-to-severe CD received EEN for 2 weeks followed by CDED+PEN (*n*=30) for up to 24 weeks, or 8 weeks of EEN followed by PEN with free diet (*n*=26). Fecal samples were collected at baseline and weeks 2, 8, 14, and 24 from patients, and at baseline from healthy family members (FM; *n*=108). Samples were subjected to 16S rRNA gene amplicon sequencing, and linear mixed models were applied; false discovery rate-adjusted *p*<0.05 was considered significant.

**Results:**

At baseline, patients with CD had microbiome signatures distinct from healthy FM, with 26 differentially abundant bacterial genera (all *p*<0.05). Two weeks of EEN induced microbiome compositional changes in both diet groups, such as reducing fibre-degrading taxa (*e.g., Bifidobacteria, Faecalibacterium; p*<0.01), effects which were maintained in the EEN group by week 8. CDED+PEN increased relative abundances of *Ruminococcus* (*p*=0.006), *Lachnospiraceae*_NK4A136_group (*p*=0.008), and *Monoglobus* (*p*=0.023) at week 8 compared to EEN. However, only CDED+PEN maintained certain taxonomic changes at weeks 14 and 24 (*e.g.,* increased uncultured *Oscillospiraceae*, *Christensenellaceae* R-7 group), suggesting sustained diet-induced alterations to the microbiome. CDED+PEN further reduced *Oscillospiraceae* UCG-005 at weeks 8 and 14 compared to healthy FM, and only patients in sustained remission at week 24 maintained lower *Oscillospiraceae* UCG-005 abundance compared to FM.

**Conclusions:**

CDED+PEN induced gut microbiota compositional alterations distinct to EEN. Despite gradual liberation of diet in the CDED+PEN group, certain gut microbiota changes were sustained in the long-term. Diet-induced microbiome alterations were linked to sustained long-term remission, potentially serving as biomarkers of positive clinical outcomes in pediatric CD. Our findings may help establish a framework for personalizing diet therapies based on the gut microbiome to optimize outcomes for pediatric patients with CD.

**Funding Agencies:**

Nestlé Health Sciences

